# Editorial: Incorporating Phase 0 microdosing as a powerful tool into a new vision of drug development

**DOI:** 10.3389/fphar.2024.1455643

**Published:** 2024-07-16

**Authors:** H. Markus Weiss, Yuichi Sugiyama, Esther van Duijn

**Affiliations:** ^1^ Novartis Biomedical Research, Inc., Basel, Switzerland; ^2^ iHuman Institute, Shanghai Tech University, Shanghai, China; ^3^ Laboratory of Quantitative System Pharmacokinetics, Pharmacodynamics, Josai International University (JIU), Tokyo, Japan; ^4^ The Netherlands Organization for Applied Scientific Research, The Hague, Netherlands

**Keywords:** Phase 0, microdosing, drug discovery and development, intra-target microdosing (ITM), exploratory clinical trials, 3R’s, modeling and simulations, vulnarable populations

Phase 0 microdosing, the testing of compounds at subtherapeutic doses in human to enable early decisions in drug discovery and development has been utilized for some years. The uptake was slow even though the concept is attractive and the pre-clinical safety package enabling first human testing is reduced (Burt et al., 2020). The attractiveness comes from the potential to facilitate better decisions informed by early human data, save animal experiments, obtain data in vulnerable populations, and increase the quality and value of subsequent studies at therapeutic doses. The new concept of intra-target microdosing (ITM) promises to enhance scope, value, and impact (Burt et al., 2017) increasing the appeal of Phase 0 studies. Technological developments, the importance of streamlining the drug development process to improve health at acceptable cost, and the increasing desire to reduce animal testing (Combes et al., 2003) give additional momentum. ITM has the capacity to assess safety or efficacy related biomarkers going beyond the earlier microdose focus on pharmacokinetics, and in combination with Positron Emission Tomography target expression or engagement related endpoints. Recently intratumoral ITM was tested as a tool for precision medicine in oncology (Peruzzi et al., 2023).

As part of this Research Topic Gundle et al. describe a technology platform for intratumoral microdosing of oncology drugs called CIVO (clinical *in vivo* oncology) platform. The platform was designed to generate human PD data early at low risk for study participants and first described by Klinghoffer et al. (2015). CIVO is based on an injectable device that can be used for surface-accessible tumors and can microdose up to eight different drugs. Gundle et al. provide an overview of this device over the years, showing its successful use for several drugs over multiple clinical trials. The platform enables the direct access to human PD data in a safe and controlled way, a clear advantage over animal efficacy data that comes with the risk of poor translation. Based on the observed intra target responses of the candidate drugs it is possible to rule-in and rule-out oncology drug candidates, develop stratification strategies, and refine indications early in the process. Current limitations such as the focus on surface accessible tumors and future perspectives like the use of the platform as companion diagnostic tool are highlighted.

Aoki et al. have developed a physiologically based pharmacokinetic model to assess which compound and target properties support or rather constrain the use of ITM to provide data related to target interaction and PD. The study revealed that ITM is capable of engaging targets at similar levels as systemically administered therapeutic doses for specific compounds. A notable decrease in the probability of relevant target interaction was observed when the predicted therapeutic dose exceeds 10 mg. The study also identified several critical factors affecting the success of ITM. These encompass both low dissociation constants (less than nM range) of drug candidates and an optimum abundance of molecular targets in the target organ.

Roffel and van Hoogdalem share their experiences with and provide an overview of the application of Phase 0 (microdose) and microtracer (a radiolabeled microdose typically combined with an unlabeled therapeutic dose) approaches in early clinical development. Perspectives on Phase 0 studies are promising as Phase 0 studies provide the potential to learn early about molecules in the target species human rather than base decisions on potentially misleading animal data. The decision to conduct a Phase 0 study needs to balance risk reduction against investment. The value of human intravenous PK and absolute bioavailability data is an important motivation. To increase the feasibility of Phase 0 studies, qualified non-GMP 14C-labeled drug material can remove the hurdle in time and investment for the development of GMP material early in a development program. Alternatively, the development of sensitive LC-MS/MS methods to avoid 14C labeling is an option. Regulatory hurdles for microdose studies are considered low and increasing worldwide AMS capacity and novel technologies facilitate their conduct. Roffel and van Hoogdalem encourage more use of Phase 0/microdose studies of different designs to increase productivity of the drug development process.

Udomnilobol et al. highlight a key challenge of Phase 0 work. Non-linearities in PK often take a long time to understand in detail. For compounds early in development the likelihood can be assessed based on *in vitro* and animal data (Bosgra et al., 2016), but the example of Dabigatran Etexilate nicely illustrates that risks or mechanisms of non-linearities may take many years on the market to crack. Therefore, the possibility of obtaining biased answers from a Ph 0 study needs to be considered. As is highlighted by Udomnilobol et al. iv administration can be an option to reduce this risk. ITM is not affected by the challenge of non-linear PK. Indeed, ITM has the capacity to test compounds which do not necessarily have drug like ADME properties. This provides an opportunity to test a mechanism of action early before a long compound selection constrained by ADME criteria.

With only four publications this article Research Topic illustrates the diversity of work to further explore and develop Phase 0 micro dosing. The potential of systemic microdosing has not yet been exhausted and technical developments are further decreasing hurdles. ITM is an emerging field with promises beyond oncology, specifically when target organs are small and/or amenable to local administration such as skin, tendons or small joints. Considering that the doses used in ITM could be considerably below the typical microdose threshold of 100 ug the question is if the regulatory requirements for testing of nano doses in human could be further reduced. Reduced technical quality and preclinical safety needs should be fully exploited to increase the potential of Phase 0 to relevantly reduce the need for animal pharmacology work and improve the drug development process by faster moving into human.
